# Supplementary material for: The Evolution and Characterization of the RNA Interference Pathways in Lophotrochozoa
Source: Genome Biol Evol. 2024 May 7;16(5):evae098. doi: 10.1093/gbe/evae098 (PMC11114477; doi:10.1093/gbe/evae098)
Supplement: evae098_Supplementary_Data [file evae098_supplementary_data.zip › Supplementary_materials.zip!/selected_miRNAs/NoGe_selected_miRNAs/mirdeep_pdf_files/NMRB01000097.1_Notospermus_geniculatus_strain_Ushimado_scaffold97,_whole_genome_shotgun_sequence_3796.pdf]

| | |
|---|---|
| Provisional ID | : NMRB01000097.1_Notospermus_geniculatus_strain_Ushimado_scaffold97,_whole_genome_shotgun_sequence_3796 |
| Score total | : 159 |
| Score for star read(s) | : 3.9 |
| Score for read counts | : 154.6 |
| Score for mfe | : 1.1 |
| Score for randfold | : 0 |
| Score for cons. seed | : -0.6 |
| Total read count | : 315 |
| Mature read count | : 299 |
| Loop read count | : 0 |
| Star read count | : 16 |

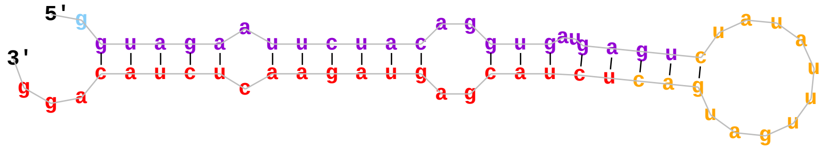


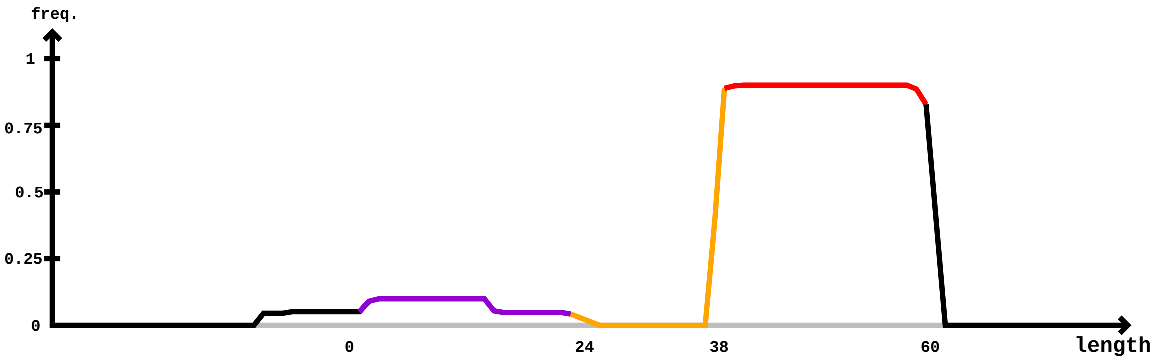


```
                                  Star                                 Mature
5'- ggugaugagucgacaguugaugacgcugcagguagaauucuacaggugaugagucuauauuugaugacucuacgaguagaacucuacagguggugacuauuacacaucgu  -3'   obs
    ggugaugagucgacaguugaugacgcugcagguagaauucuacaggugaugagucuauauuugaugacucuacgaguagaacucuacagguggugacuauuacacaucgu        exp
    ...((((.((.((.(((......((((((..(((((.((((((..(((..(((((..........))))))))..)))))).)))))..))))))))).)))).))))..  reads    mm     sample
    ....................ugacgcugcaUguagaauucuaca..................................................................  15       1      Fm2
    ...................................................................cucuacgaguagaacucuacagA....................  5        1      Fm2
    ...................................................................cucuacgaguagaacucuacagU....................  88       1      Fm2
    ....................................................................ucuacgaguagaacucuacag.....................  11       0      Fm2
    ....................................................................ucuacgaguagaacucuacagU....................  104      1      Fm2
    ....................................................................ucuacgaguagaacucuacagA....................  1        1      Fm2

    ...............................guagaauucuacaggugaugagu........................................................  9        0      Fm1
    ................................uagaauucuacaggugaugagu........................................................  2        0      Fm1
    ...................................................................cucuacgaguagaacucuaca......................  3        0      Fm1
    ...................................................................cucuacgaguagaacucuacagU....................  20       1      Fm1
    ...................................................................cucuacgaguagaacucuacagA....................  5        1      Fm1
    ....................................................................ucuacgaguagaacucuacag.....................  2        0      Fm1
    ....................................................................ucuacgaguagaacucuacagU....................  27       1      Fm1
    ....................................................................ucuacgaguagaacucuacagg....................  8        0      Fm1
    ......................................................................uacgaguagaacucuacaggA...................  1        1      Fm1

    .......................cgcugcagguagaauucuacag.................................................................  2        0      Mm2
    ................................uagaauucuacaggugaugagu........................................................  1        0      Mm2
    ...................................................................cucuacgaguagaacucuacagU....................  1        1      Mm2
    ....................................................................ucuacgaguagaacucuaca......................  1        0      Mm2
    ....................................................................ucuacgaguagaaUucuacag.....................  1        1      Mm2
    ....................................................................ucuacgaguagaacucuacagU....................  1        1      Mm2
    .....................................................................cuacgaguagaacucuacaggu...................  1        0      Mm2

    ....................................................................ucuacgaguagaacucuacagU....................  3        1      Mm1

    ...............................guagaauucuacaggugauga..........................................................  2        0      Fm3
    ...............................guagaauucuacaggugaugaguc.......................................................  2        0      Fm3
    ...................................................................cucuacgaguagaacucuacag.....................  1        0      Fm3
    ...................................................................cucuacgaguagaacucuacagU....................  4        1      Fm3
    ....................................................................ucuacgaguagaacucuacagU....................  3        1      Fm3
    .....................................................................cuacgaguagaacucuacagU....................  2        1      Fm3
```

Star Mature

ggugaugagucgacaguugaugacgcugcagguagaauucuacaggugaugagucuauauuugaugacucuacgaguagaacucuacagguggugacuauuacacaucgu

```
..................................................................cucuacgaguagaaUucuaca.....................   1   1   Mm3
..................................................................cucuacgaguagaaUucuacag....................   2   1   Mm3
..................................................................cucuacgaguagaacucuacagU...................   1   1   Mm3
...................................................................ucuacgaguagaaUucuacag....................   2   1   Mm3
```
